# Mapping of multi-elements during melting and solidification using synchrotron X-rays and pixel-based spectroscopy

**DOI:** 10.1038/srep15988

**Published:** 2015-11-02

**Authors:** E. Liotti, A. Lui, T. Connolley, I.P. Dolbnya, K.J.S. Sawhney, A. Malandain, M.D. Wilson, M.C. Veale, P. Seller, P.S. Grant

**Affiliations:** 1 Department of Materials, University of Oxford, Parks Road, Oxford OX1 3PH, UK; 2 Diamond Light Source, Harwell Science and Innovation Campus, Didcot, OX11 0DE, UK; 3 STFC, Harwell Science and Innovation Campus, Didcot, OX11 0DE, UK

## Abstract

A new synchrotron-based technique for elemental imaging that combines radiography and fluorescence spectroscopy has been developed and applied to study the spatial distribution of Ag, Zr and Mo in an Al alloy during heating and melting to 700, and then re-soldification. For the first time, multi-element distributions have been mapped independently and simultaneously, showing the dissolution of Ag- and Zr-rich particles during melting and the inter-dendritic segregation of Ag during re-solidification. The new technique is shown to have wide potential for metallurgical and materials science applications where the dynamics of elemental re-distribution and segregation in complex alloys is of importance.

The mechanical properties of many cast metallic components are dictated by the microstructure formed during the liquid to solid transformation, which often cannot be significantly altered subsequently. Although it is well-known from theory and experience that the dynamic distribution of temperature and alloying elements ahead of the growing solid/liquid interface is critical in controlling the final microstructure, such as the columnar grain to equiaxed grain transition, there are few experimental approaches that have yielded solute distribution information, especially in commercial, multi-component alloys. Early work using organic alloys, transparent analogues of metals, provided information on morphological development of the solid phase during solidification^1^,^2^,^3^, but no solute distribution information. X-ray diffraction studies of solidifying melts have provided information on phase transformations and crystal nucleation, although without morphological details^4^,^5^. Synchrotron X-ray based radiography and tomography imaging techniques have provided both solute distributions and grain morphology information in metallic systems at high temperature^6^,^7^,^8^,^9^,^10^. However, these spatial distributions of elemental concentrations rely on X-ray absorption contrast between solid and liquid phases due to the preferential segregation of one element to one or the other. Therefore semi-quantified information is generally available only for binary, or quasi binary alloys^11^,^12^,^13^,^14^. In this paper, a new synchrotron-based elemental imaging technique combining X-ray radiography and fluorescence spectroscopy is presented that overcomes this limitation. The technique is used to study dynamic changes in the distribution of Ag, Zr and Mo simultaneously as elemental powders dissolve into an Al-Cu based model alloy during heating, and then during subsequent solidification. The technique is suggested to have potential for investigating a wide range of metallurgical and materials science phenomena at elevated temperature in complex multi-component materials.

Experiments were carried out at the B16 Test beamline^15^ at the Diamond Light Source (UK) and Fig. 1(a) shows the schematic arrangement while Fig. 1(b) is an image of the furnace cell. A foil sample prepared as described below was positioned in the furnace cell with the flat, exposed surface positioned at 45 to the incoming unfiltered white X-ray beam. Conventional transmission radiographs were collected using a 0.5 LYSO scintillator coupled via a 90° turning mirror to an AVG Manta G125B CCD camera, while the fluorescence signal was magnified using a pinhole camera arrangement and collected using a HEXITEC^16^,^17^ CdTe pixellated detector module. This detector had an 80 × 80 array of 250μm pixels that was capable of collecting the X-ray fluorescence spectrum from the sample at each pixel and allowed the construction of 2D maps of several elements simultaneously. The pinhole was placed at 130mm from the sample surface and at 90° to the incoming X-ray beam, with the HEXITEC detector 2310mm from the pinhole, which provided a spatial resolution of 3.75μm/pixel and 13.75μm/pixel with field of view (FOV) of 5.1mm × 3.8mm and 1.1mm × 1.1mm for radiography and fluorescence modes respectively.

The furnace cell consisted of a single bronze plate heater with a groove to allow accurate positioning of the sample and a central hole through which the X-rays passed. The temperature of the plate was recorded by two embedded K-type thermocouples and controlled by a PID system based on National Instruments LabView software. The furnace cell was enclosed in a controlled atmosphere chamber that was flushed with a positive pressure of Ar throughout.

Samples were designed to facilitate the observation of the spatial distribution of multiple elements during either solid-state heating, melting or solidification and cooling. A Al-25wt% Cu alloy was chosen as the base for the samples because it is common in solidification studies and the preferential segregation of Cu to the liquid on solidification provides contrast for the radiographic mode. To this base alloy Ag, Zr and Mo were added: Ag and Zr are considered to dissolve reasonably quickly in molten Al whereas Mo is considered to dissolve much more slowly. Particulates of Ag as 1 mm spheres, Zr as short rods cut from 0.5 mm wire and Mo flakes cut from 0.2 mm foil were mixed and introduced as a loosely packed layer between two thicker layers of loosely packed, freshly milled Al–25 wt% Cu swarf. This layered, particulate assembly was prepared directly in a graphite die in a Ar filled glove box, and was then fully consolidated at 530 °C, 20MPa and 45 min to produce a 2cm high ×2cm diameter solid cylinder. Foil samples of 10mm × 20mm × 0.2mm for the synchrotron experiments were then sectioned from this cylinder across a diameter so that the sandwiched Ag, Zr and Mo particulate layer ran approximately across the sample width, which was polished to a 1µm finish on both sides. The foils were finally sandwiched between two BN 100µm cover plates and mounted in the furnace.

Element distributions during the initial heating and then melting cycle were studied by simultaneously recording the transmitted and fluorescence X-ray signals during heating. Note that in this particular sample and beamline combination, fluorescence maps due to Cu could not be obtained because *K*_*α*_ for Cu is 8.05KeV, which was too low to be detected due to self-absorption. Therefore in terms of interpretation, it was assumed that once melting of the Al-25 wt% Cu matrix started, at and above the Al-Cu eutectic temperature of approximately 550 °C, the Cu remained preferentially dissolved in the Al-rich liquid i.e. there were no additional reactions of the Cu with the solid Ag, Zr or Mo. On subsequent re-solidification after homogenisation of the liquid at 700 °C, it could be conceived that Cu might form complex ternary or quaternary intermetallic compounds in combination with the Al, Zr, Ag and Mo, but post-solidification microstructural analyses of the samples (e.g. of the type shown later in Fig. 4(a)) suggested that Cu behaved largely as it does in the solidification of Al-Cu binary alloys, that the presence of Mo, Zr and Ag had little effect on its solidification behaviour, and *vice-versa*.

Initially, temperature was increased at 50 °C.min^-1^ from room temperature to 540 °C and radiographs obtained at 0.125Hz. Above 540 °C, radiographs were collected at 0.5 Hz while recording the fluorescence signal in continuous mode and increasing the temperature in discrete steps of 5 °C every 5 minutes. Radiograph and fluorescence videos are shown in Video1 (uploaded as supplementary materials) and summarized in Fig. 2, which shows examples of both the transmission radiographs and the Ag, Zr and Mo fluorescence maps below and above the Al-25 wt%Cu melting temperature of approximately 550 °C. The maps were generated by plotting the intensities of the the *K*_α_ emission energy peaks within the energy range of 21 to 23 keV for Ag, 15 to 16 keV for Zr and 17 to 18 keV for Mo. In the partially molten state at 570 °C, three main phases were distinguishable within the common field of both detectors, and are labeled A, B and C in Fig. 2. The fluorescence maps show that phase A was Ag-rich, while B and C were Zr-rich; there was also Ag enrichment around B. The Mo maps were comparatively uniform with very low counts suggesting that Mo was not present in this field. As the temperature increased, Ag and Zr dissolution accelerated (Video1) so that at 660 °C, both Ag and Zr were almost completely dissolved and uniformly distributed in the Al-rich liquid. There was no significant change in the Mo maps, supporting the assumptions of its low dissolution rate in molten Al. To confirm the low and flat Mo distribution was due to restricted dissolution and not due to insensitivity of the HEXITEC detector to Mo fluorescence, a further map was obtained from a different region containing a Mo flake, as shown in Fig. 3, and indicated excellent resolution of the Ag, Zr and Mo signals simultaneously.

Assuming a homogeneous liquid was obtained after holding at 700 °C, solute re-distribution during solidification was studied by reducing the temperature in steps smaller than 1 °C with chemical maps and radiographs collected at each temperature. These experiments were more difficult to control because of convection effects in the liquid and because nucleation of solid, columnar grains occurred generally in a different place in each otherwise identical experiment. Convection was caused by the vertical arrangement of the furnace and the presence of different elements with significant differences in density (e.g. Al and Ag have densities of 2.7 and 10.5 g.cm^−3^ respectively) that induced thermo-solutal gradients in the liquid, thus creating fluctuations of the solute boundary layer at the solid-liquid interface and differences in the local dendrite tip velocity and morphology^6^.

Nonetheless, Video2 (uploaded as supplementary materials) shows the development of a dendritic array of columnar grains during solidification by radiography, along with the corresponding multi-element fluorescence maps. Figure 4(a) shows a radiograph of the microstructure after solidification had completed at 550 °C with the smaller HEXITEC field of view superimposed in red and the Ag, Zr and Mo fluorescence maps shown to the side. Dendrites grew from approximately left to right with some variation in spacing due to convective effects and some noise in the furnace control during cooling. Inter-dendritic contrast due to elemental segregation was clear in the radiographs and Fig. 4(b) shows a plot of pixel intensity, along the labelled Line 1 in the radiograph. However, amongst the various possibilities, the radiographic data were unable to provide any insight into which element(s) were segregated preferentially to this inter-dendritic region. By using a similar plot from Line 2 in the fluorescence maps (noting again Cu could not be resolved), Fig. 4(b) reveals the inter-dendritic regions to be rich in Ag and comparatively denuded in Zr and Mo–information unavailable in radiographic mode. Ag has complete solubility in liquid Al and comparatively low solubility in the solid phase and thus behaved similarly to Cu by segregating to inter-dendritic regions. Zr undergoes a peritectic reaction with liquid Al during cooling to form Al_3_Zr particulates, and several Zr-rich bright spots can be identified in the Zr map in Fig. 4(a); similarly to Zr, Mo can form Al_12_Mo or other compounds during solidification although in this case, the bright spots in the Mo map in Fig. 4(a) may be remnants of undissolved Mo flakes, given its slow dissolution during heating (post-solidification analyses using energy dispersive spectroscopy identified several undissolved Mo flakes).

The plot of Ag fluorescence counts with distance in Fig. 4(b) shows a peak-to-peak concentration spacing of 150 to 200μm in the segregation pattern, consistent with the inter-dendritic spacing suggested by the radiograph in Fig. 4(a).

In conclusion, a novel synchrotron radiography-fluorescence technique has been developed to study dynamic phenomena in alloys containing more than two elements, and has been applied for the study of the melting and re-solidification of a Al-Cu-Ag-Zr-Mo model alloy. The technique is a significant step forward in X-ray radiography, offering some of the advantages of room temperature microscopy studies of element spatial distributions, but with a high temperature, comparatively large sample volume and dynamic capability. In this initial orientation, spatio-temporal resolution was sufficient to capture some details of element diffusion and segregation during melting and solidification. However, considerable improvements are likely achievable, including improved furnace design to offer greater control of dynamic phenomena, significantly enhanced temporal resolution using a higher flux X-ray source on an undulator or wiggler beamline, and improvements in spatial resolution through modified optical arrangements. Results from these refinements will be reported later, but we conclude here that it has been shown that the combined radiography and pixel-based spectroscopy elemental imaging technique offers significant potential in the field of metallurgy and materials science to study a range of high temperature diffusion and elemental segregration phenomena.

## Additional Information

**How to cite this article**: Liotti, E. *et al.* Mapping of multi-elements during melting and solidification using synchrotron X-rays and pixel-based spectroscopy. *Sci. Rep.* **5**, 15988; doi: 10.1038/srep15988 (2015).

## Supplementary Material

Supplementary Video 1

Supplementary Video 2

## Figures and Tables

**Figure 1:**
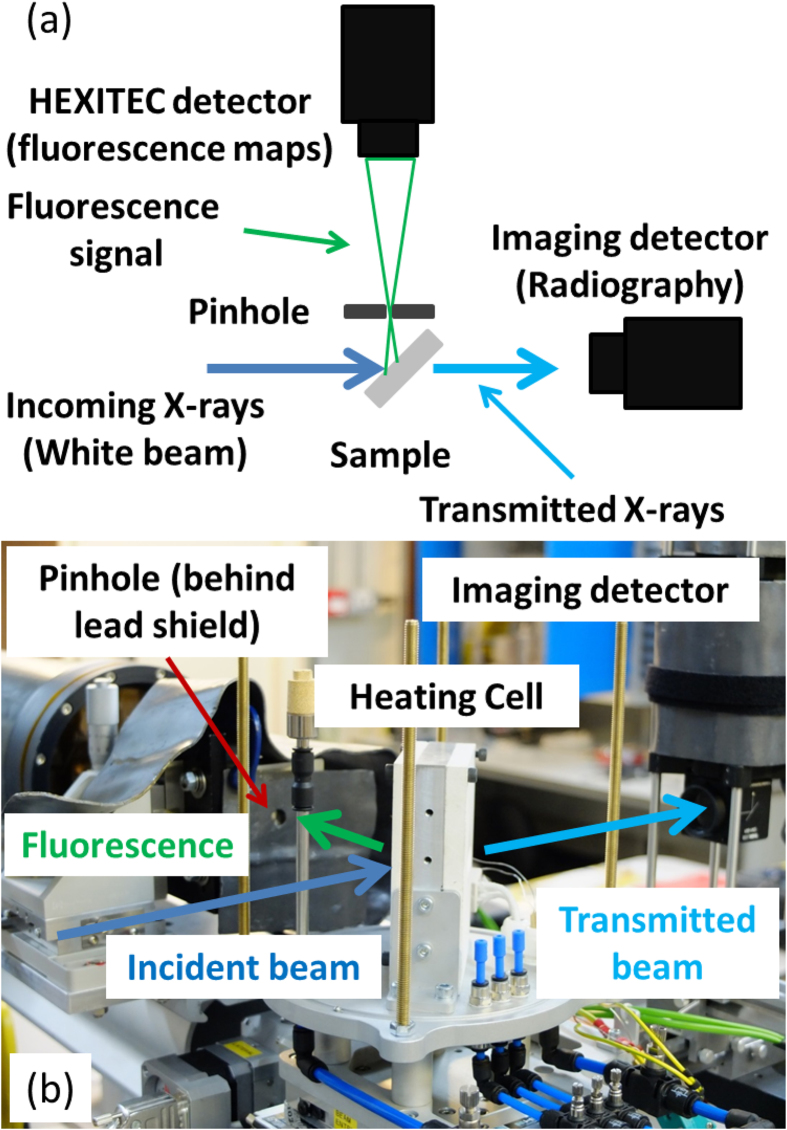
(**a**) Experimental setup (top view); and (**b**) image of the arrangement of the furnace cell, radiography camera, the pinhole and the X-ray beam path.

**Figure 2:**
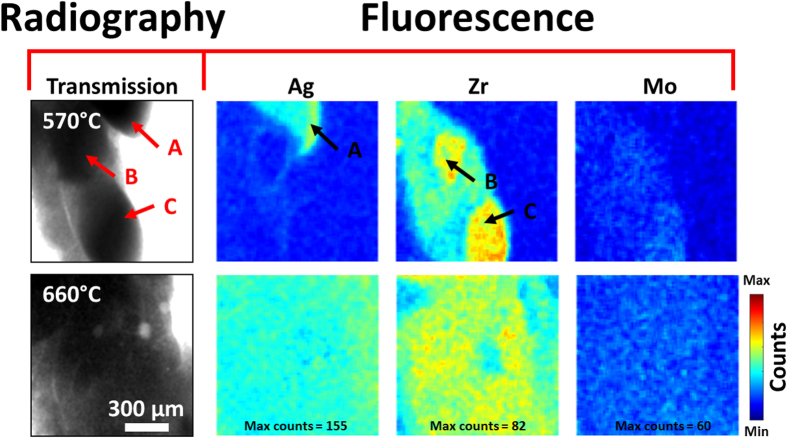
Radiograph (transmission image) and fluorescence maps taken at 570 °C and 660 °C showing Ag, Zr and Mo distributions. Three regions were detected (**A–C**) that were rich in Ag (**A**) and Zr (**B**,**C**). The maximum counts for 1 minute acquisition time were: Ag = 155, Zr = 82 and Mo = 60. The maximum counts were calculated globally for all the maps collected during the entire length of the remelting experiment. The Zr and Mo maps were corrected to take into account any overlapping of *K*_*α*_ and *K*_*β*_ peaks.

**Figure 3:**
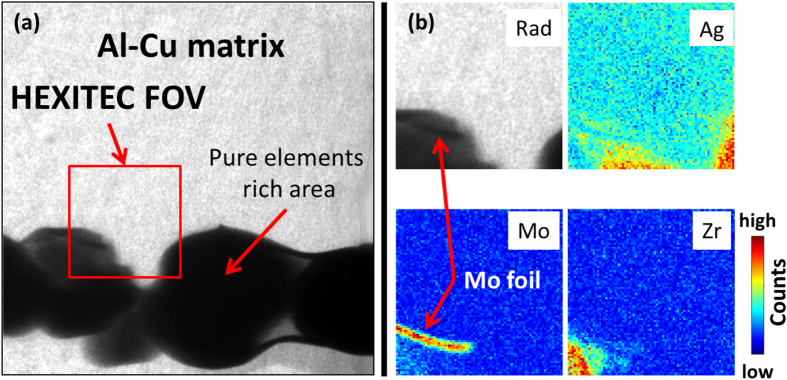
(**a**) Radiograph of a region containing Ag, Zr and Mo rich phases within the HEXITEC FOV (marked in red). In (**b**) the zoomed radiograph on the HEXITEC FOV is shown along with the Ag, Zr and Mo fluorescence maps. The Mo map reveals a distinct rich region corresponding to the undissolved Mo flake.

**Figure 4:**
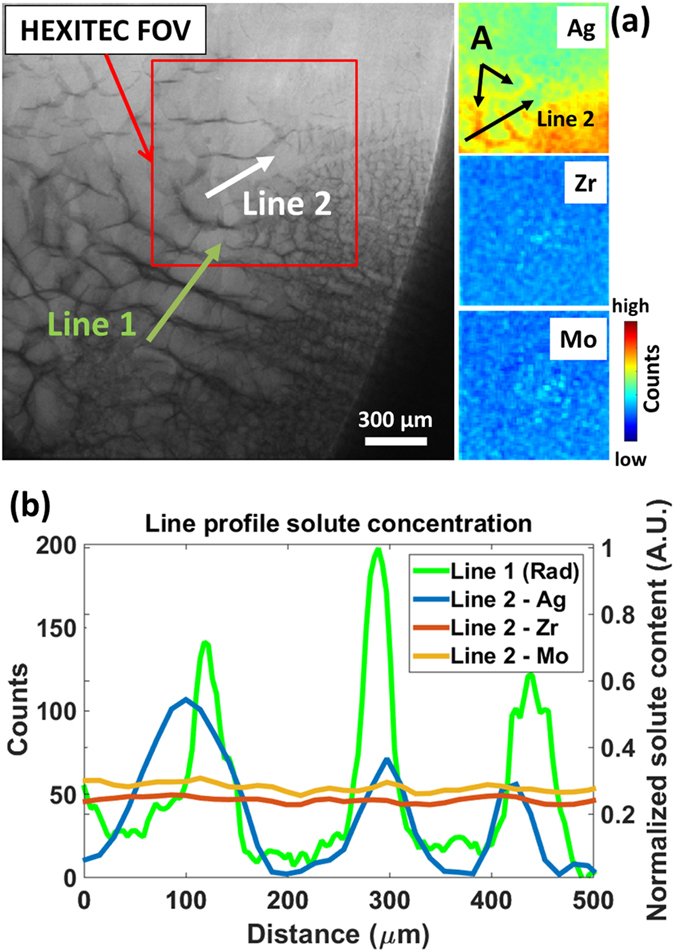
(**a**) A radiograph at 550 °C showing the re-solidified dendritic microstructure along with corresponding Ag, Zr and Mo fluorescence maps. The fluorescence field of view is shown in red. (**b**) Plots of radiograph pixel intensity (normalised to the fully mixed liquid alloy intensity) along line 1 and Ag, Zr and Mo counts from line 2 in the fluorescence maps.
